# Terrestrial Dispersal and Potential Environmental Transmission of the Amphibian Chytrid Fungus (*Batrachochytrium dendrobatidis*)

**DOI:** 10.1371/journal.pone.0125386

**Published:** 2015-04-30

**Authors:** Jonathan E. Kolby, Sara D. Ramirez, Lee Berger, Kathryn L. Richards-Hrdlicka, Merlijn Jocque, Lee F. Skerratt

**Affiliations:** 1 One Health Research Group, College of Public Health, Medical, and Veterinary Sciences, James Cook University, Townsville, Queensland, Australia; 2 Operation Wallacea, Wallacea House, Old Bolingbroke, Lincolnshire, PE23 4EX United Kingdom; 3 Sustainability Studies Program, Ramapo College of New Jersey, Mahwah, New Jersey, United States of America; 4 School of Forestry & Environmental Studies, Yale University, New Haven, Connecticut, United States of America; 5 Royal Belgian Institute for Natural Sciences, Vautierstraat 29, Brussels, 1000 Belgium; University of South Dakota, UNITED STATES

## Abstract

Dispersal and exposure to amphibian chytrid fungus (*Batrachochytrium dendrobatidis*, *Bd*) is not confined to the aquatic habitat, but little is known about pathways that facilitate exposure to wild terrestrial amphibians that do not typically enter bodies of water. We explored the possible spread of *Bd* from an aquatic reservoir to terrestrial substrates by the emergence of recently metamorphosed infected amphibians and potential deposition of *Bd*-positive residue on riparian vegetation in Cusuco National Park, Honduras (CNP). Amphibians and their respective leaf perches were both sampled for *Bd* presence and the pathogen was detected on 76.1% (35/46) of leaves where a *Bd*-positive frog had rested. Although the viability of *Bd* detected on these leaves cannot be discerned from our quantitative PCR results, the cool air temperature, closed canopy, and high humidity of this cloud forest environment in CNP is expected to encourage pathogen persistence. High prevalence of infection (88.5%) detected in the recently metamorphosed amphibians and frequent shedding of *Bd*-positive residue on foliage demonstrates a pathway of *Bd* dispersal between aquatic and terrestrial habitats. This pathway provides the opportunity for environmental transmission of *Bd* among and between amphibian species without direct physical contact or exposure to an aquatic habitat.

## Introduction

Infection by the pathogenic amphibian chytrid fungus *Batrachochytrium dendrobatidis* (*Bd*) poses a major threat to global amphibian biodiversity [1,2]. Response to infection varies considerably between species; a minority of those tested generally carry *Bd* in the absence of morbidity and may serve as aclinical reservoir hosts, such as the American bullfrog, *Lithobates catesbeianus* [3], and the African clawed frog, *Xenopus laevis* [4], whereas others are highly susceptible to chytridiomycosis and have suffered dramatic decline following introduction in wild populations [5,6]. Variation in virulence has been observed, and exposure to certain isolates of the highly pathogenic *Bd*GPL clade causes mortality in amphibians more quickly than others [7,8]. *Bd* demonstrates low host species specificity and as of 2012, infection had already been reported in 516 species from 52 countries [9], and evidence suggests this pathogen is native in some parts of its range but emerging and spreading in others [10,11]. Identified 15 years ago [12], the geographic origin and subsequent pathways of global and local *Bd* dispersal remain largely speculative, although recent studies show *Bd* is now commonly spread via the international and domestic trade in live amphibians [13–16]. However, mechanisms of dispersal outside the amphibian host and in the absence of anthropogenic assistance are more obscure.

Direct and indirect modes of *Bd* dispersal and transmission within wild amphibian populations have been postulated, but few have been demonstrated. Direct contact between animals engaged in amplexus or territorial confrontation is thought to be a common mode of transmission [17]. Contact with contaminated water is another avenue, and *Bd*'s motile uniflagellated zoospores can disperse through a water body either by swimming short distances or by being carried in water currents [18]. Waterfowl might carry *Bd* between separate water bodies, either on their feathers or feet [19–21]. However, the high prevalence of *Bd* detected in terrestrial and arboreal amphibian species that infrequently contact each other and typically do not directly engage with other species or enter permanent water bodies [22–25], suggests the presence of additional avenues of *Bd* dispersal and environmental transmission. For example Burrowes et al. [26] detected a high prevalence of infection (44.1%) in *Eleutherodactylus coqui*, a direct-developing terrestrial anuran inhabiting leaf litter in the cloud forest in Puerto Rico and McCracken et al. [27] found 33% of canopy-dwelling amphibians infected in a lowland Ecuadorian rainforest. *Bd* has also been detected on 62% of terrestrial soil-dwelling caecilians sampled in Cameroon [28,29]. Collectively, the detection of *Bd* on amphibians that inhabit the forest canopy, terrestrial leaf littler, and soil suggests a common terrestrial existence where its dispersal and transmission are not constrained by the absence of permanent water.

The spread of *Bd* through Central and South America is associated with dramatic amphibian declines and extirpations [5,6,30,31] and interestingly, affected sites include remote wilderness areas and national parks where anthropogenic-assisted *Bd* spread is expected to be minimal [31–33]. Although a wave of *Bd* appears to have swept southeast through Central America during the 1980's [10,32], relatively little is known of its present distribution and ecological impact in Honduras. Infected amphibians have been reported from two locations, Pico Bonito National Park [34] and Cusuco National Park (CNP) [24], but the country boasts a mosaic of additional montane cloud forests likely to be similarly affected, but not yet surveyed. It has been estimated that nearly 50% of 111 amphibian species in Honduras have suffered declines in recent years from a combination of factors, including chytridiomycosis, and seven endemic anuran species were believed extinct [35], although one (*Craugastor milesi*) was recently rediscovered [36]. *Bd* has been detected in Honduran terrestrial anurans that undergo direct metamorphosis in leaf litter, including *Craugastor aurilegulus* and *C*. *rostralis* [24,34], and the source of pathogen exposure to these species remains enigmatic. Similarly, *Bd*-positive terrestrial frogs have been detected in Costa Rica (*Oophaga pumilio* and *Craugastor fitzingeri*), prompting the authors to suggest that *Bd* can survive on the moist forest floor where transmission might occur [32].

Since *Bd* occurs in the superficial skin of infected metamorphosed amphibians, there appears to be potential for infectious zoospores and sporangia within shedding skin to contaminate environmental substrates. Newly post-metamorphic anurans, in particular, often exhibit both elevated *Bd* prevalence and zoospore loads [24,37–39], so their emergence from water might represent a considerable pathway of *Bd* dispersal into the terrestrial zone. To explore this potential avenue of terrestrial *Bd* spread we investigated whether terrestrial vegetation becomes contaminated with *Bd* following contact with recently metamorphosed amphibians under natural field conditions.

## Materials and Methods

### Ethics

Amphibian sampling in CNP adhered to established protocols [40] and were authorized by the Instituto Nacional de Conservacion y Desarollo Forestal Areas Protegidas y Vida Silvestre (ICF) of Honduras as part of the long-term Biodiversity Monitoring Programme performed by Operation Wallacea. Permission to export samples was granted by Honduran permit #'s 44735 and 19987.

### Study Site

This investigation was performed from 9 July to 6 August 2013 in Cusuco National Park (CNP), a montane rainforest located in the Sierra de Omoa of northwestern Honduras. The altitude of CNP ranges from 550 m to 2200 m and fieldwork was performed between 1300 m and 1600 m at three different river sites (Rio Cusuco, N 15.495, W 88.213, elev. 1600 m; Rio Cortecito, N 15.523, W 88.288, elev. 1350 m; and Rio Danto, N 15.530, W 88.277, elev. 1545 m). Previous work identified widespread distribution and high prevalence of *Bd* in CNP at these locations [24] and its presence in the region for approximately two decades or greater [41]. Recently metamorphosed individuals of four tree frog species susceptible to *Bd* were targeted for sampling (*Duellmanohyla soralia*, *Plectrohyla dasypus*, *Plectrohyla exquisita*, and *Ptychohyla hypomykter*). Of these species, *P*. *dasypus*, previously demonstrated the highest prevalence of infection both at the species level (78%) and among recently metamorphosed individuals (94%) [24]. Most sampling was performed at night when animals were more active and likely to be encountered on riparian vegetation, although some opportunistic sampling occurred in the day. Most frogs were encountered within 5 m of the water's edge, but some were found up to 50 m from the river. Sampling was restricted to frogs resting on leaves, and not those perched on stalks or branches.

### Leaf and Amphibian Sampling

**Fig 1:**
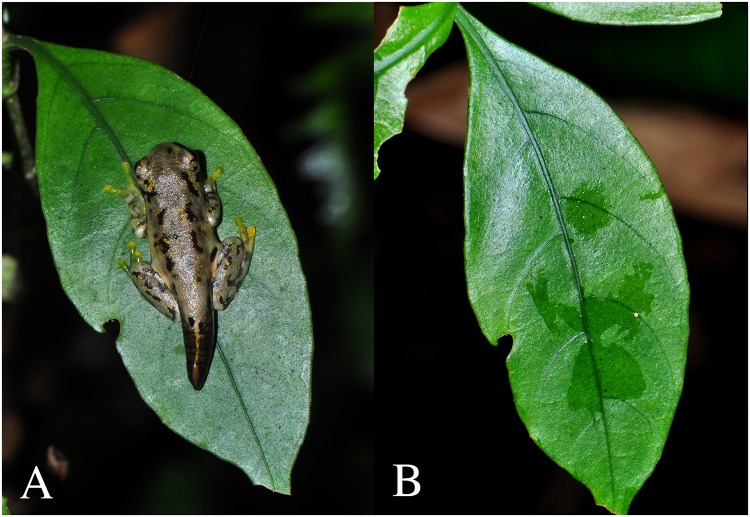
Recently metamorphosed *Plectrohyla dasypus* on terrestrial vegetation in Cusuco National Park, Honduras. (A) Amphibian as encountered on vegetation. (B) *Bd*-positive residue remaining on the leaf after amphibian removal.

Recently metamorphosed amphibians were removed from leaves by inverting them above a new plastic bag, into which the amphibian either jumped or was guided by a gentle tap on the underside of the leaf. Care was taken not to exert pressure between the frog and leaf, to prevent increased potential *Bd* shedding. Vegetation was sampled first, and then the corresponding amphibian was sampled. Nitrile gloves were worn and changed between every swab collected to reduce the risk of sample cross contamination. Leaves and frogs were each sampled with sterile fine-tipped rayon swabs (Medical Wire & Equipment Co., #MW113). For leaves, each swab was drawn across the leaf surface 20 times, where the amphibian was perched and in most instances, had left a small film of moisture visible on the leaf's surface, approximately 0.5 cm in diameter, marking the amphibians' location (Fig 1). For amphibians, the hands, feet, and pelvic patch were swabbed five times each following protocols established by Hyatt et al. [40]. Swab buds were snapped off into 2 mL microcentrifuge tubes filled with 1 mL 70% ethanol as a preservative. After sampling was completed, each amphibian was replaced to its original position in the vegetation.

### Temperature

Immediately upon encountering an amphibian perched on vegetation, the amphibian's dorsal body surface, the vegetation surface, and the air temperature were measured to characterize the environmental conditions *Bd* would be exposed to, if present. Temperatures were measured using a Raytek ST81 Non-contact Infrared Thermometer (RAYST81, emissivity set to 0.95), from a distance of 0.5 m or less. Accuracy of the thermometer is ± 1% of reading or ± 1°C, whichever is greater. This technique obtains amphibian body temperature readings within 0.5°C of cloacal temperatures [42]. Air temperature was measured with the attachable RTD temperature probe.

### Water Sampling

Water samples from rivers at the three sites were collected and filtered for *Bd* detection. These samples were processed for *Bd* testing following established protocols [43], with the exception that a peristaltic pump was used to increase the efficiency of sampling efforts by maximizing the volume of water filtered. We used sterile silicone rubber peristaltic pump tubing and replaced a new length for the collection of each sample. Water was pumped through Sterivex filter capsules (0.22 micron pore size) until the flow rate greatly diminished. Then the volume filtered was measured and recorded. The content of each filter capsule was rinsed with 50 mL phosphate buffered saline solution and then pumped dry. A bead of clay sealant was used to plug the outlet spout of the capsule before being preserved by adding 0.9 mL of Qiagen ATL tissue lysis buffer with a sterile 1 mL syringe. Luer-Lok screw caps sealed the inlet spout of the capsules and a bead of quick-drying epoxy was applied behind each clay plug in the outlet spout to provide the seal with reinforcement during transit. Fresh pairs of Nitrile gloves were worn each time a water sample was collected. All water sampling was performed during daytime hours. Water temperature was measured at the time of sampling using the attachable RTD temperature probe of the Raytek ST81 Non-contact Infrared Thermometer.

### Real-Time PCR Analysis

Samples were processed via a sensitive quantitative PCR assay (qPCR) specific to *Bd* following the established protocol [44] and with the addition of BSA to the qPCR master mix as per Garland et al. (2010) [45]. Samples were extracted with 100 μl Prepman Ultra (Applied Biosystems, California, USA), with a final 20 μl of supernatant removed for downstream use. An aliquot of this supernatant was diluted 1:10 in DNase-free water for qPCR. The qPCR protocol used SensiMix II Low Rox (Bioline, Massachusetts, USA) as the qPCR master mix [46]. For each sample, 5 μl of 1:10 dilution of swab DNA was added to each well for a final total qPCR volume of 20 μl. Samples and controls were run in triplicate with three positive, standard control samples (100, 10, and 1 zoospore/well, made from JAM81 pure culture; see Boyle et al. 2004 for standard control construction) and one non-template control (DNase free, molecular-grade water). When the qPCR assay failed to detect *Bd* in all three wells, the sample was deemed negative for *Bd*. Samples that produced a positive signal for *Bd* in either two or three wells on the first run were considered positive for *Bd*. When only one of three replicates detected *Bd*, the sample was rerun (in triplicate again) in a subsequent plate. For rerun samples that had at least a cumulative total of two of six replicates positive for *Bd* (from at least two separate plates), the sample was deemed positive for *Bd*. All zoospore loads described in this report have not been converted and reflect the actual zoospore loads present in 5 μl DNA (1:10 dilution), placed into 20 μl reaction volumes.

### Data Analysis

We applied Chi-square test on a 2x2 contingency table to determine whether row and column marginal frequencies were equal. The values in the matrix included: number of *Bd*-negative frogs associated with *Bd*-negative leaves (5), number of *Bd*-negative frogs associated with *Bd*-positive leaves (1), number of *Bd*-positive frogs associated with *Bd*-negative leaves (11), number of *Bd*-positive frogs associated with *Bd*-positive leaves (35). Analysis was performed in R (R Development Core Team 2013 version 3.0.11 using package STATS (Chisq.test; version 3.0.3).

## Results

### Amphibian Swab *Bd* Results

*Bd* was detected on 46 of 52 (88.5%) amphibians and from all four species (Table 1). The average *Bd* zoospore equivalent load detected on *Bd*-positive amphibians was 103.94 and ranged from 0.06–1,574.62.

**Table 1:** Presence of *Batrachochytrium dendrobatidis* (*Bd*) detected on amphibians and vegetation sampled in Cusuco National Park, Honduras.

Date	Sample#	Species	Site	Frog qPCR	Leaf qPCR	Frog ZSE	Leaf ZSE
Jul 9 2013	HN13BD107	*Plectrohyla dasypus*	CO	+	-	0.45	n/a
Jul 9 2013	HN13BD109	*Ptychohyla hypomykter*	CO	+	-	0.54	n/a
Jul 9 2013	HN13BD110	*Plectrohyla dasypus*	CO	+	+	24.39	17.81
Jul 9 2013	HN13BD111	*Plectrohyla dasypus*	CO	+	-	2.38	n/a
Jul 9 2013	HN13BD112	*Plectrohyla dasypus*	CO	+	+	29.19	12.25
Jul 9 2013	HN13BD114	*Plectrohyla dasypus*	CO	+	+	2.38	16.11
Jul 9 2013	HN13BD115	*Plectrohyla dasypus*	CO	+	+	3.04	14.57
Jul 9 2013	HN13BD116	*Plectrohyla dasypus*	CO	+	+	2.10	0.81
Jul 9 2013	HN13BD117	*Plectrohyla dasypus*	CO	+	+	93.05	4.70
Jul 9 2013	HN13BD118	*Plectrohyla exquisita*	CO	+	+	53.94	1040.45
Jul 9 2013	HN13BD120	*Plectrohyla dasypus*	CO	+	+	0.39	1.52
Jul 10 2013	HN13BD121	*Duellmanohyla soralia*	CO	+	-	16.75	n/a
Jul 10 2013	HN13BD122	*Plectrohyla dasypus*	CO	+	-	0.35	n/a
Jul 10 2013	HN13BD123	*Duellmanohyla soralia*	CO	-	-	n/a	n/a
Jul 10 2013	HN13BD124	*Plectrohyla dasypus*	CO	-	-	n/a	n/a
Jul 10 2013	HN13BD125	*Duellmanohyla soralia*	CO	+	+	0.64	0.34
Jul 10 2013	HN13BD128	*Plectrohyla dasypus*	CO	+	-	3.00	n/a
Jul 10 2013	HN13BD129	*Duellmanohyla soralia*	CO	-	-	n/a	n/a
Jul 10 2013	HN13BD130	*Plectrohyla dasypus*	CO	-	+	n/a	0.12
Jul 10 2013	HN13BD131	*Plectrohyla dasypus*	CO	+	+	2.00	0.64
Jul 10 2013	HN13BD132	*Ptychohyla hypomykter*	CO	+	+	28.77	8.39
Jul 10 2013	HN13BD133	*Plectrohyla dasypus*	CO	+	+	13.79	1.97
Jul 10 2013	HN13BD134	*Plectrohyla dasypus*	CO	+	+	23.76	0.93
Jul 10 2013	HN13BD135	*Plectrohyla dasypus*	CO	+	+	33.18	1.68
Jul 10 2013	HN13BD136	*Plectrohyla dasypus*	CO	+	-	2.11	n/a
Jul 10 2013	HN13BD137	*Duellmanohyla soralia*	CO	+	+	22.42	23.06
Jul 11 2013	HN13BD144	*Plectrohyla dasypus*	CO	+	+	11.82	1.07
Jul 11 2013	HN13BD145	*Plectrohyla dasypus*	CO	+	+	1085.68	43.30
Jul 14 2013	HN13BD161	*Duellmanohyla soralia*	CO	+	-	0.06*	n/a
Jul 14 2013	HN13BD164	*Ptychohyla hypomykter*	CO	+	+	660.54	49.09
Jul 15 2013	HN13BD166	*Plectrohyla dasypus*	CO	-	-	n/a	n/a
Jul 15 2013	HN13BD170	*Plectrohyla dasypus*	CO	+	+	236.29	139.30
Jul 15 2013	HN13BD171	*Duellmanohyla soralia*	CO	+	+	1.20	0.47
Jul 15 2013	HN13BD172	*Plectrohyla dasypus*	CO	+	+	58.50	0.79
Jul 15 2013	HN13BD173	*Plectrohyla dasypus*	CO	+	+	57.69	4.38
Jul 15 2013	HN13BD174	*Plectrohyla dasypus*	CO	+	+	17.44	40.30
Jul 15 2013	HN13BD175	*Plectrohyla exquisita*	CO	+	+	38.66	1.00
Jul 15 2013	HN13BD177	*Plectrohyla dasypus*	CO	+	+	18.10	0.32
Jul 15 2013	HN13BD178	*Plectrohyla exquisita*	CO	+	+	281.79	10.93
Jul 15 2013	HN13BD179	*Ptychohyla hypomykter*	CO	+	+	0.64	0.25
Jul 15 2013	HN13BD180	*Ptychohyla hypomykter*	CO	+	-	1.37	n/a
Jul 15 2013	HN13BD181	*Duellmanohyla soralia*	CO	+	+	4.06	3.36
Jul 16 2013	HN13BD183	*Plectrohyla dasypus*	CO	+	+	1574.62	3.90
Jul 16 2013	HN13BD249	*Ptychohyla hypomykter*	CO	+	+	39.07	0.23
Jul 18 2013	HN13BD261	*Plectrohyla dasypus*	DA	+	+	147.26	7.84
Jul 14 2013	HN13BD323	*Plectrohyla dasypus*	DA	-	-	n/a	n/a
Aug 5 2013	HN13BD389	*Plectrohyla exquisita*	CU	+	-	27.10	n/a
Aug 5 2013	HN13BD390	*Plectrohyla exquisita*	CU	+	+	34.02	0.44
Aug 5 2013	HN13BD391	*Plectrohyla exquisita*	CU	+	+	48.13	1.97
Aug 6 2013	HN13BD407	*Plectrohyla dasypus*	CU	+	+	25.23	0.29
Aug 6 2013	HN13BD408	*Plectrohyla exquisita*	CU	+	+	1.03	2.63
Aug 6 2013	HN13BD409	*Plectrohyla exquisita*	CU	+	-	52.55	n/a

Survey sites include Rio Cortecito (CO), Rio Danto (DA), and Rio Cusuco (CU). Average zoospore equivalent (ZSE) per qPCR reaction is reflected for all *Bd*-positive samples. Asterisk denotes the single sample that produced a positive reaction in 2/6 wells; all other samples produced *Bd*-positive reactions in 2/3, 3/3, or 3/6 wells.

### Leaf Swab *Bd* Results

*Bd* was detected on 36 of 52 (69.2%) leaves, 97.2% of which had a *Bd*-positive recently metamorphosed amphibian on them (35/36) (statistical significance of the association, df = 1, chi-squared = 6.23, p-value = 0.013) (Table 1). Only one *Bd*-positive leaf had an amphibian that tested *Bd*-negative. The average *Bd* zoospore equivalent load detected on *Bd*-positive leaves was 40.48 and ranged from 0.12–1,040.45.

### River Water Filter *Bd* Results

The presence of *Bd* was detected in all three river water samples (Table 2). The average *Bd* zoospore equivalent load per liter of river water was 0.23 and ranged from 0.03–0.57. Daytime water temperature averaged 17.0°C and ranged from 16.3–17.5°C.

**Table 2:** Presence of *Batrachochytrium dendrobatidis* (*Bd*) detected in water filter samples collected from amphibian survey sites in Cusuco National Park, Honduras.

Sample#	Site	Vol (ml)	T (°C)	ZSE/L
HN13W01	CO	11000	17.5	0.08
HN13W02	DA	2700	17.1	0.03
HN13W03	CU	4600	16.3	0.57

Survey sites include Rio Cortecito (CO), Rio Danto (DA), and Rio Cusuco (CU). Volume of water filtered, water temperature, and average *Bd* zoospore equivalent (ZSE) per liter of river water is reflected for all samples.

### Amphibian and Vegetation Temperatures

Most animals were sampled during nocturnal surveys, from 20:00–2:00 hrs (n = 45), although some were occasionally encountered and sampled during the day, from 10:45–15:00 hrs (n = 7). Night temperatures of the frogs' dorsal surfaces, leaf surfaces, and air averaged 17.0°C, 17.1°C, and 16.9°C and ranged from 15.2–18.9°C, 15.8–19.1°C, and 15.3–17.8°C, respectively, whereas day temperatures averaged 20.8°C, 21.0°C, and 20.2°C and ranged from 18.4–26.6°C, 18.8–7.0°C, and 19.4–21.9°C, respectively.

## Discussion

We frequently detected *Bd* on leaf surfaces after removal of recently metamorphosed *Bd*-positive frogs, indicating their emergence does contribute towards the spread of *Bd* from aquatic into terrestrial locations. Average zoospore loads detected on leaf surfaces were comparable to those from corresponding amphibian skin swabs, and sometimes greater. The presence of *Bd* on riparian vegetation allows exposure to occur in the absence of direct physical contact with *Bd*-positive animals or contaminated water. Accordingly, this pathway of *Bd* dispersal and terrestrial exposure provides one possible explanation for the source of infection previously detected in amphibians that do not demonstrate a strong association with water.

**Fig 2:**
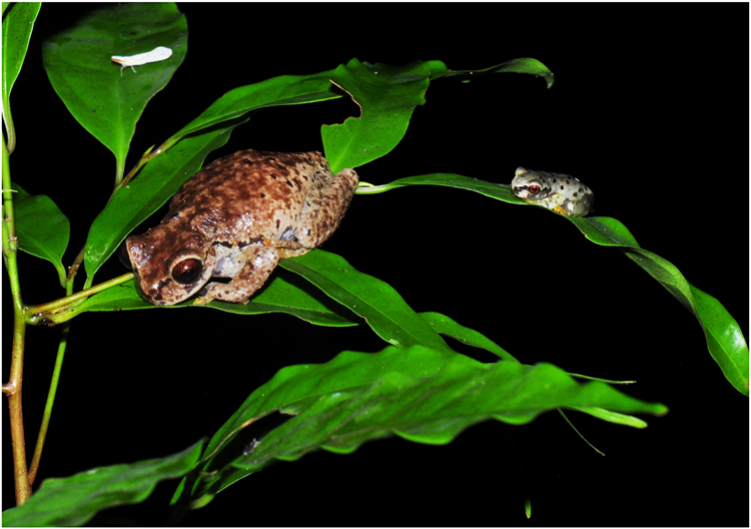
Adult and recently metamorphosed *Plectrohyla dasypus* resting in close proximity in Cusuco National Park, Honduras. The skin swab collected from this juvenile (HN13BD145) tested positive for *Bd* infection and exhibited a considerable zoospore load (1,085.68 ZSE), as did the leaf swab (43.30 ZSE), demonstrating the risk of exposure to the nearby *Bd*-negative adult through contact with contaminated vegetation.

This pathway of *Bd* spread may occasionally facilitate transmission between aquatic and terrestrial species and from juvenile to adult frogs, if foliage maintains infectious *Bd* loads. On 11 July 2013, both a recently metamorphosed and adult *Plectrohyla dasypus* were observed perched together on the same plant at the same time, approximately 5 cm apart (Fig 2). The skin swab sample collected from this juvenile frog (HN13BD145) exhibited a considerable zoospore load (1,085.68), as did the leaf swab (43.30), demonstrating a high risk of exposure to the nearby adult which tested *Bd*-negative at the time of sampling. Following metamorphosis, this species leaves the aquatic habitat and moves into arboreal vegetation, reducing the likelihood of subsequent *Bd* exposure from contaminated river water. The high prevalence of infection in *P*. *dasypus* juveniles detected in this and previous surveys [24] suggests that their seasonal emergence *en masse* may release a substantial quantity of *Bd* into the riparian zone shared with amphibians that approach the water's edge, but do not typically enter it.

Although we identified a potential mechanism of pathogen exposure to terrestrial amphibians, the role of contaminated vegetation in *Bd* transmission remains in question. Detection of *Bd* via qPCR indicates DNA presence, but does not reveal condition at the time of sampling. A lack of experimental work to evaluate the persistence and detectability of *Bd* DNA following cell death makes it difficult to discern whether we likely detected viable *Bd* or instead DNA fragments from expired cells that continued to react with *Bd* qPCR primers. This interpretation limitation is not exclusive to environmental *Bd* swab samples, but likewise applies to amphibian skin swabs; a positive qPCR result does not independently demonstrate the viability of *Bd* on that animal. Still, environmental conditions observed at all sampling localities in CNP were similar to those in the laboratory where *Bd* survived outside a host [19] and may aid persistence of *Bd* on leaf surfaces. Temperatures recorded in the field were all within or near the range for optimal in vitro growth of *Bd* (17–25°C) and well below its thermal maximum of 28°C [47], although optimal temperature regimes may vary between *Bd* isolates [48] and none from Honduras have yet been characterized. Desiccation poses the other well-defined abitoic limitation to *Bd* survival [19,49] but the presence of both high relative humidity and a dense forest canopy preventing direct sun exposure is correlated with higher *Bd* prevalence and infection loads [50]. These conditions are typical of CNP, a montane cloud forest, and expected to prolong drying. Lastly, laboratory experiments have shown that when maintained under suitable temperature and moisture levels (and without bacteria), *Bd* can survive in the absence of a host for at least two months in water or moist sand [19]. Thus, additional laboratory work is needed to test the survival times of cultured *Bd* on leaf surfaces to identify the potential duration of this form of environmental persistence and evaluate the average *Bd* loads needed to cause successful transmission under naturalistic conditions.

Previous efforts to illustrate environmental *Bd* transmission have mainly focused on exposure to permanent water bodies inhabited by *Bd*-infected amphibians [18,19]. Laboratory trials demonstrated transmission of *Bd* between experimentally-infected and uninfected tadpoles of *Rana muscosa* and also from tadpoles to post-metamorphic animals, when occupying a shared water source [18]. Successful transmission required a 2–3 week duration of exposure, likely impeded by dilution of the pathogen in a naturalistic environment, similar to the low densities of *Bd* detected in the water samples collected at our survey sites in CNP (Table 2). To encourage transmission after short-term exposure, laboratory experiments have often employed highly concentrated inoculates of approximately 100 million *Bd* zoospores delivered in less than 100 mL of water [51–53] whereas the highest concentration detected in a natural body of water has been 3 million zoospores L^-1^ and less than 100 zoospores L^-1^ is common [54]. In this context, the concentrated *Bd* loads we detected on leaf surfaces in CNP relative to the adjacent *Bd*-positive river water suggests that contact with affected foliage might pose a greater threat of exposure and transmission to terrestrial amphibians than would a splash of water from these rivers.

We detected the presence of *Bd* on vegetation in the understory, but periods of heavy rain are expected to also flush *Bd* into the soil and leaf litter below. Surveys in CNP have identified the presence of live aquatic crustaceans (copepods and ostracods) inhabiting terrestrial water films on forest floor leaf litter [55,56], suggesting moisture persistence in this limnoterrestrial habitat. The persistence of these water films in humid rainforest environments would help protect *Bd* from desiccation in a seemingly terrestrial habitat, and also allow exposure to amphibians that occupy leaf litter and burrow into the ground. Accordingly, this mode of *Bd* dispersal and indirect exposure may explain the origins of infection documented in species of soil-dwelling salamanders [22,23,25] and caecilians [28,29].

Numerous biologic and abiotic factors are expected to influence the frequency of *Bd* dispersal from aquatic into terrestrial habitats and potential consequences. The prevalence and intensity of *Bd* detected in amphibian populations often demonstrates fluctuations due to seasonal changes in environmental conditions and these factors will affect the amount of zoospores available to be shed into the terrestrial environment [49,54,57]. Rowley et al. [58] investigated the presence of *Bd* in terrestrial retreat sites of two aquatic stream frog species (*Litoria lesueuri* and *L*. *nannotis*), and did not detect *Bd* in 122 environmental swab samples. As suggested by the authors, the observed *Bd* absence may have been influenced by the low prevalence and infection loads concurrently detected in the adult amphibians sampled at these locations. Our results show that in a locality where both *Bd* prevalence and infection loads are high, it is common for *Bd* to be shed into terrestrial locations, including amphibian retreat sites.

The presence of *Bd* in terrestrial habitats should be considered when identifying potential threats to amphibian species of concern. Although it has been suggested that *Bd* poses the greatest risk of infection to amphibians breeding in permanent streams [59], we caution against this generalization and encourage additional surveillance in terrestrial and arboreal amphibian habitats where animals continue to test positive for *Bd*, despite pathways of exposure being more obscure. The frequency of *Bd* exposure from terrestrial substrates is unknown but may be considerable where optimal environmental conditions are present, especially if it can survive as a saprobe as previously suggested [12]. An improved understanding of *Bd* dispersal and persistence in the natural environment is essential to better explain and predict the continued spread of this pathogen in regions where the anthropogenic-assisted exposure to *Bd*-positive amphibians or substrates is unlikely.
